# Effect of sublingual immunotherapy on platelet activity in children with allergic rhinitis^☆^

**DOI:** 10.1016/j.bjorl.2016.03.006

**Published:** 2016-04-22

**Authors:** Yanqiu Chen, Lifeng Zhou, Yan Yang

**Affiliations:** a Guangzhou Medical College, Guangzhou Women and Children's Medical Center, Department of Otolaryngology, Guangzhou, China; b Sun Yat-sen University (Northern Campus), School of Public Health, Department of Nutrition, Guangzhou, China

**Keywords:** Allergic rhinitis, Platelet activation, Sublingual immunotherapy, Platelet Factor-4, Beta-Thromboglobulin, Rinite alérgica, Ativação plaquetária, Imunoterapia sublingual, Fator 4 plaquetário, Beta-tromboglobulina

## Abstract

**Introduction:**

The role of platelet activation in allergic inflammation is receiving increasing attention. Sublingual immunotherapy for allergic rhinitis can modify the immunological process to an allergen, rather than simply treating symptoms.

**Objective:**

The aim of this study was to explore the role of platelet activation during sublingual immunotherapy in children with allergic rhinitis.

**Methods:**

Forty-two House Dust Mite – sensitized children with allergic rhinitis were enrolled and received House Dust Mite allergen extract for sublingual immunotherapy or placebo. Serum of different time points during treatment was collected and used for detection of Platelet Factor-4 and Beta-Thromboglobulin concentration by Enzyme-Linked Immuno Sorbent Assay.

**Results:**

Our data showed decreased expression of Platelet Factor-4 and Beta-Thromboglobulin protein after one year's sublingual immunotherapy. In addition, the decrease of symptom scores and serum Platelet Factor-4 and Beta-Thromboglobulin protein concentrations was positively related.

**Conclusion:**

During sublingual immunotherapy, platelet activation was inhibited significantly. Our results might indicate that inhibition of platelet activation within the systemic circulation is an important mechanism during sublingual immunotherapy.

## Resumo

**Introdução:**

O papel da ativação de plaquetas na inflamação alérgica recebeu atenção crescente. A imunoterapia sublingual para rinite alérgica pode modificar o processo imunológico a um alergeno, em vez de tratar os sintomas simplesmente.

**Objetivo:**

O objetivo deste estudo foi explorar o papel da ativação plaquetária durante a imunoterapia sublingual em crianças com rinite alérgica.

**Método:**

Quarenta e duas crianças com rinite alérgica sensibilizadas por Ácaros de Poeira Domiciliar foram inscritas e receberam extrato de alergeno de Ácaros de Poeira Domiciliar para imunoterapia sublingual ou placebo. O soro de diferentes pontos no tempo durante o tratamento foi recolhido e utilizado para a detecção de fator 4 plaquetário e concentração de beta-tromboglobulina por Ensaio Imunoenzimático.

**Resultados:**

Nossos dados mostraram diminuição da expressão de fator 4 plaquetário e proteína beta-tromboglobulina após imunoterapia sublingual de um ano. Além disso, a diminuição dos escores de sintomas e o fator 4 plaquetário sérico e concentrações de proteína beta-tromboglobulina foram relacionados de maneira positiva.

**Conclusão:**

Durante imunoterapia sublingual, a ativação plaquetária foi inibida significativamente. Os nossos resultados podem indicar que a inibição da ativação de plaquetas dentro da circulação sistêmica é um mecanismo importante durante imunoterapia sublingual.

## Introduction

Sublingual immunotherapy (SLIT) is the only treatment that regulates the immunological process during development of allergic rhinitis (AR), rather than simply treating symptoms.^1^, ^2^ However, the underlying mechanisms in the process and potential biomarkers are still not fully characterized.

Platelet activation occurs during antigen-induced airway reactions in allergic and asthmatic subjects. Raised levels of platelet-derived mediators, such as the chemokines, Beta-Thromboglobulin (BTG) and Platelet Factor-4 (PF4), are observed in plasma and bronchoalveolar lavage fluid of atopic individuals. These mediators have the ability to activate eosinophils, increase expression of Fc-IgG and Fc-IgE receptors, and stimulate basophils to release histamine.^3^, ^4^, ^5^, ^6^ Since SLIT can inhibit allergic inflammation significantly, we postulate that SLIT may affect platelet activation in AR children.

In the current study, we aimed to clarify the effect of SLIT on platelet activation of AR children by detecting changes of serum PF4 and BTG concentration.

## Methods

### Patients

Forty-two children aged 6–12 years with a clinical history of House Dust Mite (HDM) induced AR for at least one year were enrolled. Skin Prick Test (SPT) was performed to screen children allergic to HDM. Those with chronic diseases (e.g. asthma, malnutrition, anatomic malformation of the respiratory system, chronic lung disease, heart disease, gastro-oesophageal reflux disease, cystic fibrosis) and those with a history of chronic drug use (e.g. oral or nasal corticosteroids, antiepileptics, immune suppressives) were excluded from the study. The study was performed with the approval of local ethics committee and with the parent's written informed consent.

### Sublingual immunotherapy and grouping

The HDM allergen extract for SLIT was manufactured by Wolwopharma Biotechnology Company (Zhejiang, China) and used in the form of drops (n° 1, 1 mg/mL; n° 2, 10 mg/mL; n° 3, 100 mg/mL and n° 4, 333 mg/mL). According to the manufacture's instruction, the patients were asked to take increasing doses (from n° 1 to n° 3) during the first three weeks’ up-dosing phase, and then were instructed to take 3 drops of n° 4 solution once daily during the maintenance phase. Drops were instructed to be kept under the tongue for 2–3 min before swallowed. Children in the placebo group received diluents containing 50% glycerol and 50% saline buffer. All the children were grouped as SLIT (21 children) and placebo (21 children) group randomly. The drugs were labeled with patient code numbers, and the investigator assigned patients in a sequential randomized fashion to a study code number. Individual drug bottles were identity masked in order that both patients and researchers were blind to treatment assignment. Study blinding was preserved at the study sites until all subjects completed the study. Compliance with medications was assessed both by the parent questionnaire and by measurement of drug weight administered every second week.

### Symptom scores

The nose symptoms (runny nose, sneezing, blocked nose, itching nose) were scored by the children with the help of parents. A score of 0 was defined as none (no symptoms present), a score of 1 was defined as mild (mild symptoms that do not interfere with any activity), a score of 2 was defined as moderate (slightly bothersome symptoms that slightly interfere with activity/nighttime sleep), and a score of 3 was defined as severe (bothersome symptoms that interfere with activity/nighttime sleep).^7^, ^8^ The scores were recorded every morning daily for at least 12 weeks and then were averaged.

### Blood samples preparation and analysis

The blood samples from children were collected between 11 am and 2 pm by veinpuncture method. Serum was acquired after coagulation of the blood for 1–2 h at room temperature and centrifuged at 3000 × *g* for 15 min at 4 °C. Serum samples were stored at −80 °C and used for Enzyme-Linked Immunosorbent Assay (ELISA). Total protein concentration was determined with Bio-Rad protein assays according to Bradford. ELISA kits (Diagnostica Stago, France) were used for measuring serum PF4 and BTG concentration.

### Statistical analysis

All data were expressed as mean ± SD. Statistical significances between different groups were determined using nonparametric Mann–Whitney *U* test. The Spearman rank correlation test was used to analyze the correlation between symptom score and PF4 or BTG concentration; *p* < 0.05 was considered as significant difference.

## Results

### Demographic characteristics of study population and clinical outcome

This study was conducted with 42 children, 21 of whom enrolled in SLIT group, with ages ranging between 72 and 144 months (mean age: 120.7 ± 44.0 months, 10 males), and 21 of whom enrolled in placebo group with ages ranging between 75 and 141 months (mean age: 123.0 ± 42.3 months, 11 males). The age, sex, duration of disease, baseline symptom scores between two groups were comparable without significance. The SLIT treatment was effective and the symptoms scores decreased significantly compared with both placebo group and baseline symptom scores (Table 1).

**Table 1:** Demographic characteristic of 42 AR children.

	SLIT group	Placebo group
*Number*	21	21
*Sex (male/female)*	10/11	11/10
*Age (months)*	120.7 ± 44.0	123.0 ± 42.3
*Duration of disease (year)*	5.2 ± 2.4	6.1 ± 3.7
*Total IgE (kU/L)*	465 ± 226	513 ± 316
*HDM specific IgE (kU/L)*	45 ± 21	35 ± 18

*Baseline symptoms*		
Runny nose	2.5 ± 0.3	2.1 ± 0.2
Sneezing	1.9 ± 0.1	1.7 ± 0.1
Blocked nose	2.1 ± 0.2	1.8 ± 0.3
Itching nose	1.5 ± 0.1	2.0 ± 0.4
Total score	8.2 ± 0.6	7.9 ± 0.5

*End-point symptoms*		
Runny nose	1.5 ± 0.2^a^^,^^b^	2.2 ± 0.3
Sneezing	1.4 ± 0.3^a^^,^^b^	1.5 ± 0.1
Blocked nose	1.2 ± 0.1^a^^,^^b^	1.8 ± 0.1
Itching nose	1.1 ± 0.2^a^^,^^b^	2.1 ± 0.2
Total score	5.2 ± 0.3^a^^,^^b^	7.8 ± 0.2

a Compared with placebo group, *p* < 0.05.

b Compared with baselines symptom, *p* < 0.05.

### Decreased serum PF4 and BTG protein levels during SLIT treatment

**Figure 1:**
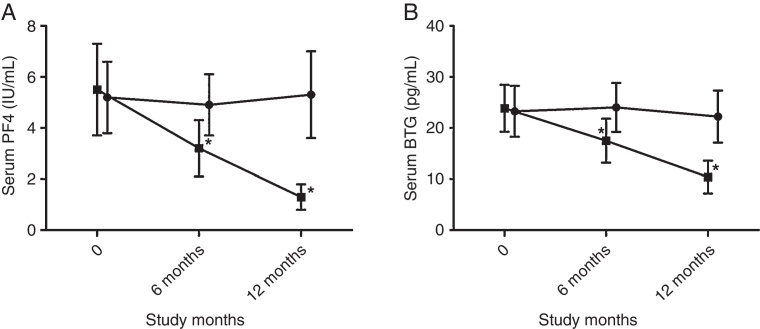
Serum PF4 and BTG protein expression decreased after 6 months’ SLIT compared with control group and baseline level with significance. The decrease maintained at least one year without rebound (*p < 0.05, comparison between two groups and baseline level; ●, represents for placebo group; ■, represents for SLIT group).

The serum PF4 and BTG protein expression during SLIT treatment were significantly lower than those in placebo group after 6 months’ treatment (PF4 3.2 ± 1.1 vs. 4.9 ± 1.2 IU/mL; BTG 17.4 ± 4.3 vs. 23.2 ± 5.1 IU/mL) and this trend was maintained for at least 1 year (PF4 1.3 ± 0.5 vs. 5.3 ± 1.7 IU/mL; BTG 10.5 ± 3.2 vs. 21.6 ± 4.8 IU/mL) (Fig. 1A and B).

### Relationship between symptom scores and serum PF4 and BTG protein levels

**Figure 2:**
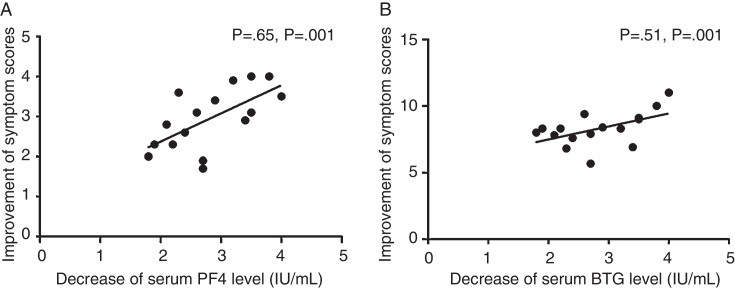
Positive relationship between improvement of symptom scores and decrease of serum PF4 and BTG protein levels of children after one year's SLIT in active treatment group (A and B).

To explore the effect of platelet activation on symptom score, we analyzed the relationship between symptom scores and serum PF4 and BTG protein levels in SLIT group. After one year of treatment, the improvement of symptom scores was positively related to decrease of serum PF4 and BTG protein levels (*p* = 0.65; *p* = 0.001; *p* = 0.51; *p* = 0.002) (Fig. 2).

## Discussion

Platelets may play an important role in the allergic inflammatory process because they are a rich source of biologically active materials capable of inducing or augmenting allergic inflammatory responses.^9^, ^10^, ^11^, ^12^, ^3^ Such materials have been demonstrated to be stored in alpha granules, which are chemokines such as PF4 and BTG. However, the role of platelets activation in SLIT was not clear, especially in children.

Our results showed that PF4 and BTG level decreased significantly after 6 months’ SLIT, and the improvement maintained at least one year. Despite decreased PF4 and BTG level observed after 3 months’ treatment, no statistically significant changes were found. Moreover, the improved symptom scores after one year SLIT was positively related with decreases of PF4 and BTG level. These results suggested that SLIT may modify the allergic process by inhibiting platelet function, at least partially.

Consistent with our results, Kowal^13^ observed increased intravascular platelet activation in House Dust Mite allergic asthma patients undergoing bronchial allergen challenge. The relationship between the changes of platelet activation markers and the development of late asthmatic response suggests that platelet activation within the circulation is critical for the development of chronic allergic inflammation. Also, Knauer^14^ has demonstrated significant changes in PF4 level of patients with pollen-induced asthma after bronchial provocation to ragweed extract.

In Alicja's study,^15^ they found that plasma PF4 level in the patient off–pollen season was significantly lower as compared with the season and did not differ significantly as compared to the healthy subjects. Conversely, in their two other studies,^16^, ^17^ the results suggested that PF4 and BTG levels were not significantly different between the patients and healthy subjects after immunotherapy. Notably, the immunotherapy in their two studies lasted only 6 months and blood sample was collected immediately after maximum dose injection. In our study, the SLIT treatment lasted at least one year and the serum was sampled at different time points. We found that decrease of serum PF4 and BTG level lasted at least one year. The difference between our and Alicja's study suggest the importance of maintenance period in immunotherapy. However, a small number of patients is one of limitations in our study. Future studies with large sample is needed to further clarify the role of PF4 and BTG in SLIT. In addition, the change of PF4 and BTG in nasal lavage before and after SLIT should be discussed in future to explore the local effect of platelet activation in mechanism of AR and SLIT.

## Conclusion

In conclusion, our study was the first to show the changes in platelet activity in vivo in AR children during SLIT. Our results might indicate that inhibition of platelet activation within the systemic circulation is an important mechanism during SLIT. SLIT may improve symptoms by inhibiting platelet activation partially.

## Ethical standards

The authors assert that all procedures contributing to this work comply with the ethical standards of the relevant national and institutional guidelines on human experimentation and with the Helsinki Declaration of 1975, as revised in 2008.

## Conflicts of interest

The authors declare no conflicts of interest.
